# Discrepancies in tuberculosis burden estimates: North Korean defectors vs. official reports

**DOI:** 10.3389/fpubh.2025.1545628

**Published:** 2025-05-30

**Authors:** Inho Jang, Heesang Han, Seungcheol Lee, Hyeongyeong Jeong, Rugyeom Lee, Yedham Kang, In-Hwan Oh, Seung Heon Lee

**Affiliations:** ^1^College of Medicine, Korea University, Seoul, Republic of Korea; ^2^Department of Preventive Medicine, Kyeong Hee University School of Medicine, Seoul, Republic of Korea; ^3^Division of Pulmonology, Sleep and Critical Medicine, Department of Internal Medicine, Korea University Ansan Hospital, Ansan, Republic of Korea; ^4^Department of Preventive Medicine, University of Ulsan College of Medicine, Seoul, Republic of Korea; ^5^Ansan Central Internal Medicine, Ansan, Republic of Korea

**Keywords:** tuberculosis, National Health and Insurance Service Claims Data, North Korean defectors, South Koreans, extrapulmonary tuberculosis, multidrug-resistant tuberculosis

## Abstract

**Objectives:**

North and South Korea have taken different approaches to tuberculosis (TB) epidemic control after the Korean War. This study aimed to compare TB epidemiology in North Korean defectors (NKDs) based on South Korean National Health Insurance (NHIS) data and assess its implications for understanding TB prevalence in North Korea.

**Methods:**

We used the NHIS claims data from 2007 to 2019 to evaluate TB epidemics in NKD and the age-and-sex matched South Korean control group. The number of participants was 35,620 for defectors and 107,016 for the control group.

**Results:**

The prevalence of TB among NKDs decreased from 466/100,000 persons in 2010 to 95/100,000 persons in 2019, while the North Korean TB prevalence as per the World Health Organization (WHO) report remained approximately 500/100,000 persons. The NKD TB prevalence was 3–7 times higher than that in the South Korean population. Additionally, the distribution of TB cases in NKDs showed distinct age-related patterns, with peaks in the 25–34 and 65 + age groups. The proportion of extrapulmonary TB in NKDs was 36–46%, similar to South Korean patterns. The estimated and reported multidrug-resistant TB rates in NKDs were higher than in the control group, highlighting potential underreporting in North Korean data.

**Conclusion:**

There were large gaps in TB prevalence between NKD and native North Korean residents and between the estimated and reported TB burden within North Korea. These findings underscore the need for targeted TB control strategies that address both health system disparities and the integration of NKDs into local healthcare services.

## Introduction

Tuberculosis (TB) is the leading cause of death among communicable infectious diseases worldwide (1). However, the estimated TB burden and reported TB epidemiology are different from those of countries in terms of the medical infrastructure, diagnostic capacity, comorbidity, and age distribution of the population (2). Moreover, the appearance of drug-resistant TB, including extensively drug-resistant (XDR) TB, and the diagnostic challenge of extrapulmonary TB in immunocompromised groups are causing concerns, especially in under-developed countries (3, 4). Therefore, in well-developed countries, the influx of TB from immigrants and refugees could be an important issue from the perspective of National Tuberculosis Control Program because TB develops in socially vulnerable groups, not to mention medically vulnerable groups such as human immunodeficiency virus (HIV) patients and malnourished people (5).

TB mortality was estimated to be 16.5 per 100,000 persons in 1926 before the Korean War but quickly increased to 350 per 100,000 persons in 1954 after the war (6, 7). During the Japanese colonial period, tuberculosis infection rates were higher in urban areas than in rural areas and higher in northern regions than in southern regions. According to a 1942 survey, the tuberculosis infection rates in North Hamgyong Province and North Pyongan Province were 71.5 and 70.5%, respectively, whereas South Gyeongsang Province and South Jeolla Province reported lower rates of 48.3 and 53.7%, respectively (6), indicating a significantly higher prevalence in the northern regions.

After the division of North and South Korea, South Korea and North Korea took different paths. In 1947, North Korea established tuberculosis sanatoriums to oversee the prevention and treatment of the disease. By 1949, North Korea had established a domestic pharmaceutical factory and, from 1960 onward, began producing isoniazid and para-aminosalicylic acid (6). In South Korea, the National Tuberculosis Center was established in 1954. However, as 90% of the national budget was allocated to military expenditures, tuberculosis control efforts could not be expanded. Despite these challenges, the South Korean government, in collaboration with the World Health Organization (WHO) and United Nations, implemented a five-year tuberculosis control plan in 1955, focusing on prevention and treatment. Subsequently, a structured tuberculosis control system was established through public health centers, which was further strengthened by the Tuberculosis Prevention Act in 1968 (8).

The TB incidence in South Korea (2) has decreased from 168/100,000 in 1990 to 29/100,000 in 2022, while that in North Korea had a higher value of 513/100,000 in 2021, according to WHO (1, 7). In South Korea, National Tuberculosis Control Program is making efforts to attain the TB goal through aggressive latent TB infection management and immigrant TB control. However, due to regional and institutional disparities and information, North Korean defectors often face challenges in accessing healthcare services. Therefore, various systems are being implemented to improve North Korean defectors’ access to healthcare. For example, the Settlement Support Center for North Korean defectors (NKDs) established a direct medical partnership with Seoul Metropolitan Seobuk Hospital in 2019 to improve healthcare accessibility for NKDs and facilitate their stable integration into society.’ However, despite these efforts, there may still be difficult access to accessibility.

The number of refugees, displaced persons, and other persons of concern to the United Nations High Commissioner for Refugees was estimated at more than 26 million in 1996 (9). North Korea is a seclusive country with few connections with other countries (10, 11). Since credible academic research is limited, we have poor understanding of the actual TB epidemic status in North Korea. NKDs may provide some insight into the TB epidemic status although they do not necessarily represent the general North Korean population due to potential selection bias.

In North Korea, it was reported that the rate of Bacillus Calmette-Guérin (BCG) vaccination which can prevent TB meningitis under the age of 19, was approximately 60% in 1998 (12), and the reported rate of drug-resistant TB is as high as 22 per 100,000 persons (13). In a published article from North Korea, atypical treatment combined with traditional Koro-medicine, such as snake venom (14, 15), reflects the desperate efforts in North Korea for TB treatment despite limited resources. Therefore, information on the recent credible TB status of North Korea is urgently needed, justifying the indirect up-to-date assessment from NKDs.

In this study, we aimed to provide sufficient information about the TB epidemic status of North Korea for effective TB control for our peninsula, analyzing the data of North Korean defectors to prepare for future unification.

## Materials and methods

We used the National Health and Insurance Service (NHIS) claims data from 2007 to 2019 to evaluate the TB epidemic situation in North Korean defectors and a control group from the general South Korean population. To protect the privacy of NKDs and ensure data security, we used deidentified data provided by the National Health Insurance Service. The number of participants was 35,620 for the North Korean defectors and 107,016 for the controls. The control group was extracted from the general South Korean population by age-sex matching with NKDs, with the number of participants being three times that of defectors.

Patients with TB were defined as those who received treatment and drug prescriptions under TB disease codes (Supplementary Table 1), for which the International Classification of Disease 10th revision (ICD-10) code was applied. Latent TB infection is classified separately under disease codes and was not included in the study population. Among the TB patients who were selected from the age-sex matched subjects, MDR-TB cases were defined by ICD-10 codes (U84.30, U84.31), which means the patients with TB resistant to at least isoniazid and rifampicin, and the prevalence of MDR TB was calculated and presented. Patients were classified into three categories, namely, “all TB patients,” “patients with TB main disease,” and “patients with TB main disease and concurrent prescription.” The case of “all TB patients” means the cases in any of the main or accompanying TB ICD-10 codes, and this is the broadest definition of TB prevalence. “Patients with TB main disease” means that only the case of the main disease as TB is included. Finally, “patients with TB main disease and concurrent prescription’ is the narrowest definition, and it includes the cases which only have TB main ICD-10 code accompanying the relevant medication history. Since diagnosis is made on individuals who had no medical utilization under a primary TB diagnosis code for the first three years following their registration in the NHIS, it is not possible to determine whether the infection occurred before, during, or after defection. The rates per 100,000 persons were used throughout the study to compare the two groups. The SAS Enterprise Guide 7.1 (Cary, NC) tool provided by the NHIS was used for descriptive data analysis. The descriptive study included age, sex, and NHIS registration year of NKDs.

As the official reference data for comparison, results from the WHO TB reports from 2000 to 2021 were also used. The Korea University IRB approved this study [Institutional Review Board (IRB no-2022AS0066)].

## Results

### Epidemic characteristics of NKDs and general control population

As presented in Table 1, the number of notified NKDs steadily increased from 11,050 in 2007 to 35,620 in 2019, and the number of female NKDs was 1.8–2.1 times higher than that of males. The number of age-sex matched South Korean control group was approximately 100,000 throughout the whole period, and the ratio of male-to-female was 1:2, comparable to that of NKDs. The age group with the highest proportion among NKDs has shifted from the 35–44 to 45–54 years age group since 2018. The proportion of the younger age group below 44 years decreased, but that of the older age group over 45 years increased (Supplementary Figure 1). The mean age in both the NKDs and South Korean control group was 36.5 years, without a significant statistical difference between the two groups.

**Table 1:** General characteristics of data from NHIS.

	South Korean control group		North Korean defectors			South Korean control group	North Korean defectors	
	Male	Female	Male	Female	*p* value	Total	Total	*p* value
Year	Total (TB)	Total (TB)	Total (TB)	Total (TB)		mean age	mean age	
2007	27,664 (27)	65,472 (30)	3,968 (12)	7,082 (11)	*p* > 0.05	28.7 ± 15.7	30.1 ± 17.8	*p* > 0.05
2008	28,446 (24)	66,232 (32)	4,840 (16)	9,453 (24)		29.2 ± 15.9	30.4 ± 17.6	
2009	29,258 (23)	67,066 (31)	5,645 (36)	11,327 (43)		29.7 ± 16.2	30.6 ± 17.6	
2010	30,035 (23)	67,876 (32)	6,516 (44)	13,427 (49)		30.2 ± 16.5	31.1 ± 17.6	
2011	30,848 (18)	68,762 (30)	7,661 (44)	15,676 (55)		30.7 ± 16.8	31.3 ± 17.8	
2012	31,620 (15)	69,485 (23)	8,490 (41)	17,259 (59)		31.1 ± 17.1	31.6 ± 17.9	
2013	32,213 (16)	70,145 (32)	9,056 (41)	18,432 (37)		31.7 ± 17.3	32.1 ± 18.1	
2014	32,773 (13)	70,734 (26)	9,595 (29)	19,817 (49)		32.3 ± 17.5	32.7 ± 18.1	
2015	33,214 (12)	71,266 (20)	10,041 (32)	20,865 (35)		33.0 ± 17.6	33.2 ± 18.1	
2016	33,590 (12)	71,674 (25)	10,428 (24)	22,065 (43)		33.7 ± 17.8	33.8 ± 18.1	
2017	33,912 (12)	71,958 (20)	10,761 (19)	23,118 (39)		34.4 ± 17.9	34.5 ± 18.1	
2018	34,169 (13)	72,199 (15)	11,021 (23)	24,078 (19)		35.2 ± 18.0	35.2 ± 18.1	
2019	34,435 (8)	72,581 (22)	11,196 (14)	24,424 (20)		36.0 ± 18.1	36.0 ± 18.1	

NHIS, National Health and Insurance Service; TB, tuberculosis.

### Comparison of TB prevalence/incidence between native South Korean populations and NKDs

**Figure 1:**
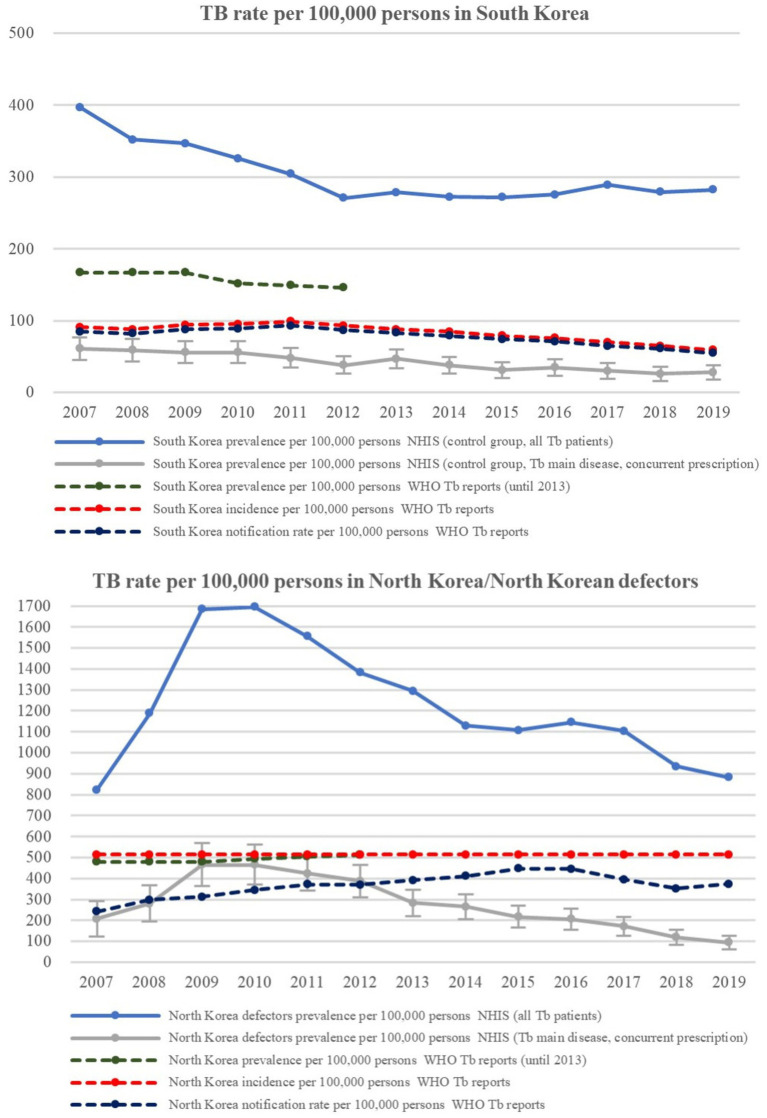
TB Prevalence/incidence of native South Korean population and North Korean residents/North Korean defectors. TB, tuberculosis; WHO, world health organization.

As presented in Figure 1, the TB prevalence among “all TB patients” from the control group in South Korea decreased from 326 per 100,000 persons in 2010 to 282 per 100,000 persons in 2019. TB prevalence among control group “patients with TB main disease and concurrent prescription” decreased from 56 per 100,000 persons in 2010 to 28 per 100,000 persons in 2019. According to official data, the TB notification rate from the WHO reports was 89 per 100,000 persons in 2010 and then steadily decreased to 55 per 100,000 persons in 2019. The TB prevalence among control group “patients with TB main disease and concurrent prescription” according to the NHIS data showed a pattern similar to the WHO TB notification rate, so it could be inferred that the data from the South Korean control group adequately represent the general epidemiological features of tuberculosis in South Korea.

Among NKDs, the TB prevalence in “all TB patients” and the “patients with TB main disease and concurrent prescription” sharply increased to 1700 per 100,000 persons and 466 per 100,000 persons in 2010 but decreased to 882 per 100,000 and 95 per 100,000 persons in 2019, respectively. However, according to official WHO data of North Korean residents, the estimated TB prevalence and incidence remained at approximately 500 per 100,000 persons for the entire period, implying that the tuberculosis epidemic among NKDs is somewhat different from the official estimates of North Korean residents. TB rate values of North Korea were 3–7 times higher than those of South Korea for each counterpart set of data.

The proportion of bacteriologically confirmed TB among new/new and relapse pulmonary TB patients in South Korea increased from 55% in 2007 to 80% in 2019, whereas that in North Korea was approximately 50% throughout the study period (Supplementary Figure 2).

### Comparison of TB prevalence between native South Korean populations and NKDs according to age group

**Figure 2:**
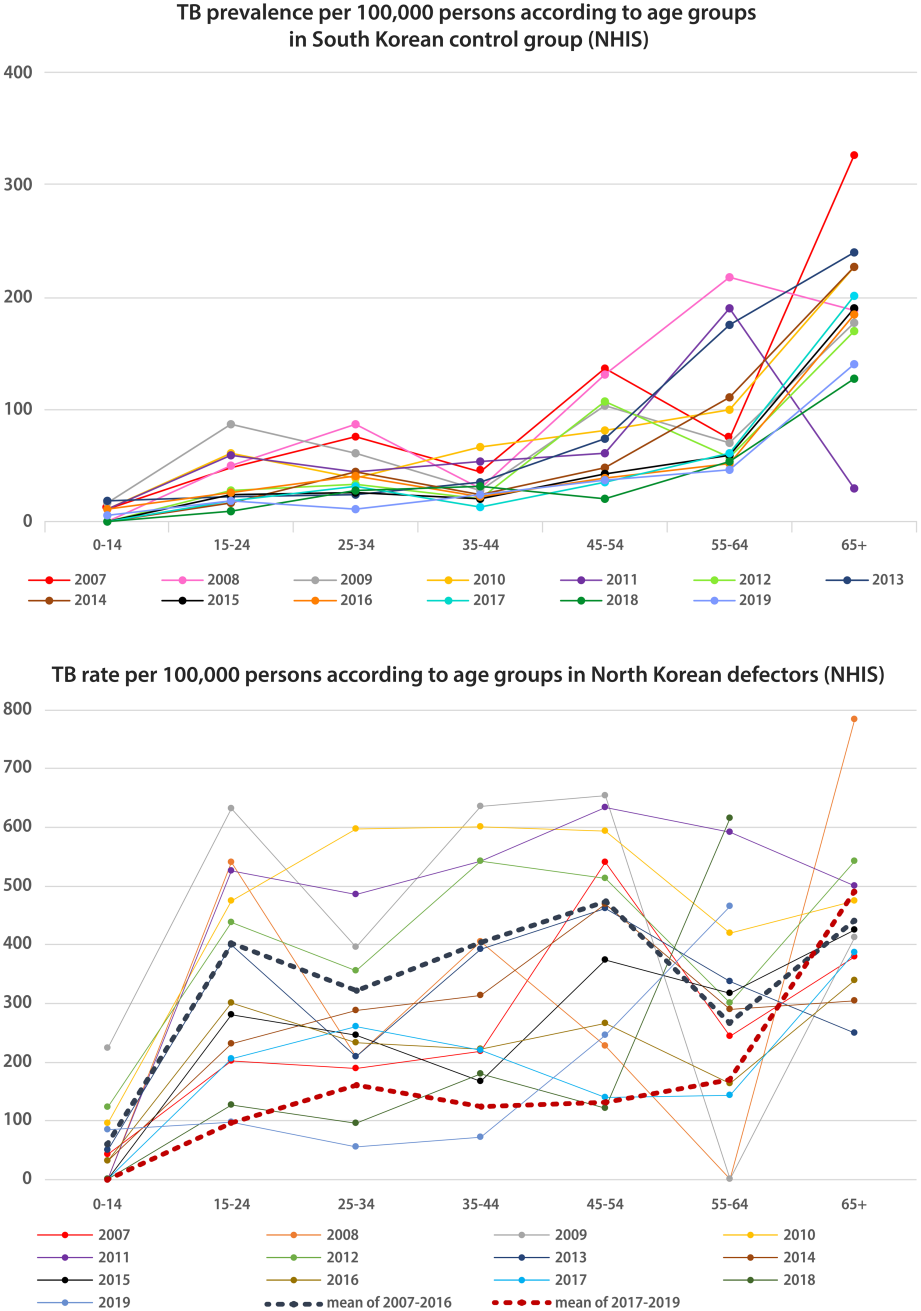
TB Prevalence/incidence according to age groups in native South Korean population and North Korean defectors. TB, tuberculosis; NHIS, national health insurance service.

As presented in Figure 2, the pattern of TB prevalence in South Korea according to age groups of patients had double peaks in the 25–34 years and 65 years-and-above age groups. However, the pattern changed into a single peak pattern, with the highest prevalence in the 65 years-and-above group since 2017. The pattern of TB prevalence among NKDs has shown triple peaks in the 15–24, 45–54, and 65-and-above age groups, but since 2017, the pattern has shown double peaks in the 25–34 years and 65 years-and-above age groups.

### Comparison of extrapulmonary TB between native South Korean populations and NKDs

**Figure 3:**
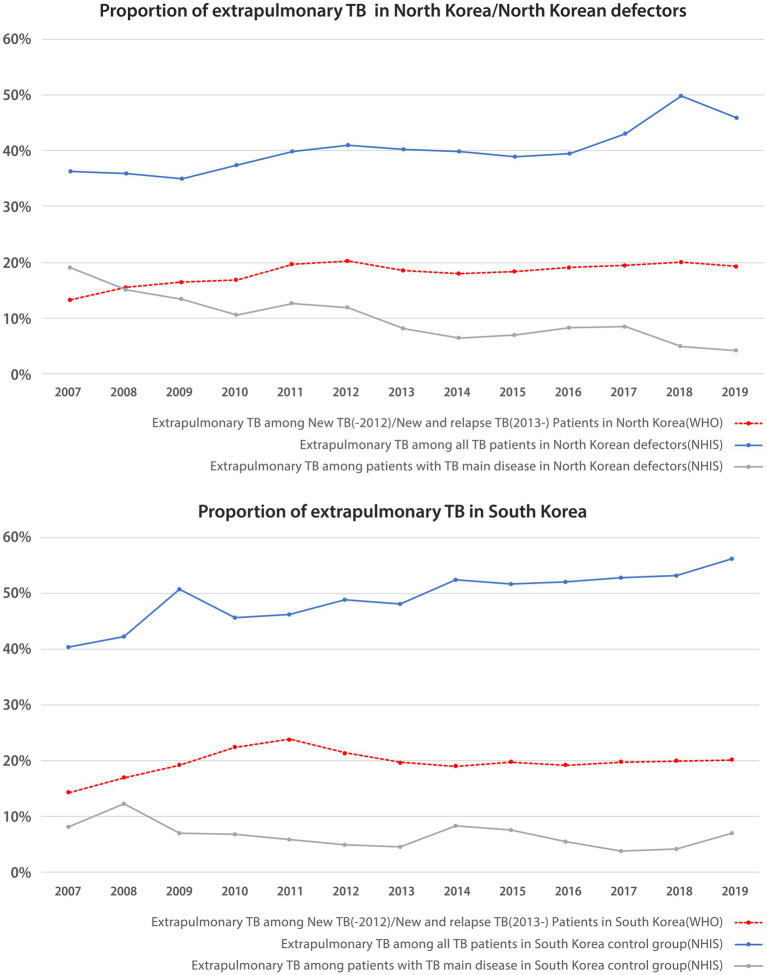
Proportion of extrapulmonary tuberculosis in native South Korean population and North Korean defectors. TB, tuberculosis; NHIS, national health insurance service.

As presented in Figure 3, in the South Korea control group, the proportion of extrapulmonary TB among “all TB patients” increased from 41% in 2007 to 56% in 2019, and that among “patients with TB main disease” fluctuated between 5 and 12%. In NKDs, the proportion of extrapulmonary TB among “all TB patient” increased from 36% in 2007 to 46% in 2019, and that among the “patients with TB main disease” decreased from 19% in 2007 to 4% in 2019. According to the WHO reports, the proportion of extrapulmonary TB in South Korea ranged from 14% in 2007 to 20% in 2019; similarly, in North Korea, it ranged from 13% in 2007 to 19% in 2019. Therefore, the general proportion levels and patterns of extrapulmonary TB did not appear to differ much between South and North Korea.

### Comparison of multidrug-resistant (MDR)-TB between native South Korean populations and NKDs

According to the NHIS data, the percentage of MDR-TB in “all TB patients” in the entire period was approximately 3%, in both the South Korean control group and NKDs.

**Figure 4:**
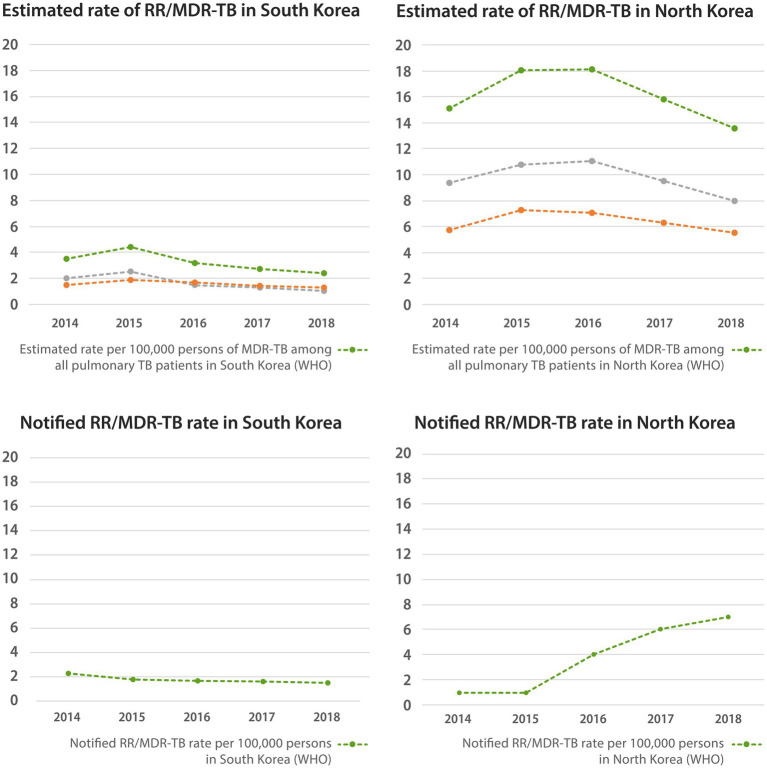
Estimated and notified rate of multidrug-resistant tuberculosis in native South Korean population and North Korean residents. WHO, world health organization; RR/MDR-TB, rifampin resistant/multidrug-resistant tuberculosis.

As presented in Figure 4, according to the WHO reports, the estimated rifampicin-resistant (RR)/MDR-TB rate among all patients with pulmonary tuberculosis in South Korea peaked in 2015 with a value of 4.4 per 100,000 persons, which then decreased to 2.4 per 100,000 persons in 2018. The estimated rates of new and retreat pulmonary tuberculosis also changed similarly (1.3 and 1.1 per 100,000 persons in 2018). The notified RR/MDR-TB rate gradually decreased from 2.3 per 100,000 persons in 2014 to 1.5 per 100,000 persons in 2018. The percentage of those who tested for RR/MDR-TB among new patients with pulmonary tuberculosis was very high, exceeding 100% in 2014, and has remained approximately 80–90% since then. In addition, 2017–2020 data on the percentage of confirmed drug resistance cases among available drug susceptibility test (DST) results from South Korea showed that isoniazid resistance and RR/MDR-TB were present in 8–9% and 0.1–0.3% of new TB patients, respectively. For DST results from retreat TB patients, the percentage of both isoniazid resistance and RR/MDR-TB was approximately 0.1–0.3%. Meanwhile, the proportion of XDR-TB among all notified pulmonary TB cases in South Korea peaked by 0.6% in 2012 and decreased to 0.2% by 2020.

In North Korea, the estimated RR/MDR-TB rate among all patients with pulmonary TB peaked in 2016, with a value of 18.1 per 100,000 persons, and gradually decreased to 13.5 per 100,000 persons in 2018. The estimated rates of new and retreat pulmonary TB also showed similar patterns, reaching 5.5 and 8.0 per 100,000 persons in 2018, respectively. The notified RR/MDR-TB rate gradually increased to 7 per 100,000 persons in 2018. The percentage of RR/MDR-TB tests among new pulmonary TB patients was virtually zero, and that among retreated TB patients increased since 2011, reaching 24% in 2020. According to the 2018 data showing confirmed resistance cases among available DST test results of retreat TB patients, 84% had isoniazid resistance and 74% had RR/MDR-TB. In addition, 2018 data on the percentage of confirmed drug resistance cases among available DST results from North Korea showed that isoniazid resistance and RR/MDR-TB were present in 84 and 74% of retreat TB patients, respectively. The percentage of RR/MDR-TB testing in North Korea was much lower than that in South Korea, and the rate of RR/MDR-TB was much higher than that in South Korea.

## Discussion

Our study based on the NHIS data showed that the TB prevalence in NKDs was higher than that in the South Korean control group, and TB was most prevalent in age groups of 20s and over 40s in NKDs, with little difference in the proportion of extrapulmonary tuberculosis between the two groups. However, there is a large gap in TB prevalence between NKDs and native North Korean residents.

### Accuracy and reliability of the WHO data for TB prevalence and incidence in North Korea

In the WHO TB reports presented in Figure 1 (red dotted line in Figure 1), the North Korean TB incidence rate per 100,000 persons was fixed at 513 per 100,000 persons throughout all recorded years. Various studies have shown that the system for the identification and control of TB in North Korea has not been functioning properly for the last 30 years (16, 17), as there has been a lack of clear estimation methods for TB incidence. Moreover, as the current post-2015 estimated incidence is based on a single prevalence survey, the accuracy and reliability of the existing data for TB prevalence and incidence in the WHO reports are highly questionable (18). According to the 2015 WHO report of the joint monitoring mission, North Korea implemented the Directly Observed Treatment (DOT) program starting in 2003 and has received support for tuberculosis control programs from the Global Fund. These efforts have had a positive impact on TB management since 2010. Also, since NKDs are enrolled in Medical Aid and do not pay health insurance premiums, TB treatment is financially accessible for them. This may have contributed to the declining TB prevalence among NKDs after 2010 (19).

A useful contextual comparison can be made between North Korea and the Philippines, both classified as high tuberculosis burden in Asia. According to WHO data, the TB incidence in the Philippines increased from 290 per 100,000 in 2009 to 638 per 100,000 in 2022—comparable to the consistent estimate of 513 per 100,000 for North Korea. However, unlike North Korea, the Philippines has a well-structured and decentralized TB control system. The National TB Control Program is led by the Department of Health and operates through a network of over 3,000 microscopy centers and 488 GeneXpert diagnostic sites. TB diagnosis and treatment are integrated into primary care, hospitals, workplaces, NGOs, and even correctional facilities, ensuring broad population access and active case detection (20). In contrast, TB control in North Korea is hindered by the lack of consistently proficient culture and DST laboratories, with GeneXpert tests used only in a very limited capacity (21). While both countries have high burdens of TB, the markedly different public health infrastructures and diagnostic capacities suggest that the officially reported TB rates in North Korea may be underestimated. However, a recent TB report in collaboration with the WHO Drug Sensitivity Laboratory Set by the Stanford university and the North Korean Ministry of Public Health suggests that efforts to improve the accuracy of TB epidemiology are underway (22, 23).

### Discrepancy between TB prevalence of NKDs from NHIS data and TB prevalence and incidence in North Korea from WHO data

The discrepancy between the TB prevalence of NKDs and the officially reported TB prevalence in North Korea might be attributed to the regional difference of TB epidemics in North Korea, given that TB notification rates for the Yanggang province (322/100,000) are lower than the total average rate (394/100,000) (24). In addition, the discrepancy could be attributed to the relatively better health status of NKDs compared with native North Korean residents (25–28), considering that the shifting of the TB prevalence distribution curve to the older age group, as presented in Figure 2, is similar to the changing curve patterns of South Korea to developed country. Moreover, improved general health conditions from previous transit living conditions could be a contributing factor (25, 26). The WHO report results were found to be in between the widest and narrowest definitions, which means that existing WHO reports may have been underestimated, considering the characteristics of the age group of NKDs. However, considering the scarcity of international reports on the national health status of North Korea, the implication of this discrepancy between NKDs and native North Korean residents should be further investigated using more reliable data.

### Extrapulmonary TB proportion of NKDs

There was little difference in the proportion of extrapulmonary TB between the South Korean control group and the NKDs. Since extrapulmonary TB cases with co-existing pulmonary TB are reported as pulmonary TB cases, it is possible that extrapulmonary TB with co-existing pulmonary TB in NKDs could have been underestimated in the NHIS data (18). The elevated proportion of extrapulmonary TB among NKDs may reflect a state of lowered immunity (29), potentially due to chronic malnutrition, high levels of psychological stress, and limited access to health care before and during migration. This observation aligns with global refugee and asylum seeker TB data. Refugees and asylum seekers in various contexts showed a higher risk of both active and extrapulmonary TB (30). The similarity between the NKDs and global refugee populations in terms of displacement-related stressors supports the plausibility of immune suppression as a contributing factor to the higher extrapulmonary TB rates. Manifestations of extrapulmonary TB in North Korean residents and defectors, including TB meningitis, which is the most common and serious type of extrapulmonary TB in North Korea (15) and is thought to be especially prevalent and fatal in children and young people (15, 31, 32), must also be evaluated.

### Lack of precise data for multidrug resistant tuberculosis in North Korea

Because information on MDR-TB in North Korean residents and defectors as well as in South Korean residents is limited, we could not present data on MDR-TB based on the NHIS study data. Instead, we showed the data collected from the scattered WHO data, including the results of the subnational drug resistance surveys in 2014. We also referenced the reports on MDR-TB treatment activities published by the Eugene Bell Foundation to access more first-hand information about drug-resistant TB among North Korean residents and showed that among TB patients selected for DST, MDR-TB was present in more than 75% of tested persons, similar to the official WHO percentage for North Korea (21, 33, 34). One concern is the successful treatment of MDR-TB. According to the WHO reports, in North Korea, the success rate of MDR-TB treatment decreased from 86% in 2012 to 75% in 2018, and the success rate of XDR-TB treatment was 79% in 2017 (Supplementary Figure 3) (18). However, in 2012, it was reported that the treatment success rate for MDR-TB patients admitted to the Eugene Bell Foundation facilities was 71%, lower than that reported in North Korea (33). Therefore, accurate data, including well-organized treatment outcomes regarding MDR-TB, are urgently needed in North Korea as well as in South Korea.

The official percentage of MDR-TB in North Korea is similar to that of South Korea, possibly because North Korea did not have a national policy and algorithm for guaranteeing universal access to DST until 2018. Therefore, DST is more likely to have been performed selectively, suggesting an underestimation of actual MDR-TB without DST results. Since North Korea seems to have established an institutional platform for universal access to DST in 2019, reliable data with wider availability are expected (18).

### Limitations of this study

Despite these results, our study has several limitations. First, patients with TB were identified through the NHIS records of treatments and drug prescriptions under defined TB disease codes without confirming the existence of the disease through actual clinical records. Due to the nature of NHIS record, the absence of pre-defection data and comorbidities remains our limitation. To overcome this weakness, multiple definitions of TB prevalence were used for sensitivity analysis. However, there could be some biases such as selection bias, confounding bias, and information bias, utilizing secondary healthcare databases without definite clinical validations. Future studies incorporating a more detailed analysis of comorbid conditions would enable a clear comparison of TB incidence patterns between two countries. Second, the number of patients with TB was generally small in each assessed year, and TB data for the post-2019 period were not included. The small size of the group may have reduced the reliability and generalizability of the study results. Also, Although North Korean defectors are originally from North Korea, they exhibit unique demographic characteristics—such as a higher proportion of females and a relatively younger age distribution. Therefore, our findings should be interpreted in the context of these characteristics, especially in light of the distinct features of tuberculosis in North Korea. Third, as NHIS data for MDR-TB are scarce, WHO reports on MDR in South and North Korea had to be used, which failed to provide sufficient information regarding the exact number of drugs with resistance and the epidemiology of XDR-TB. The definition of NKDs is based on data from the NHIS, but there may be inaccuracies in the data. To improve the quality of the TB epidemiology study in future, refining the algorithm by incorporating both diagnostic and treatment codes must be considered, because microbiologic TB confirmation could be delayed until the AFB cultures after 2 months of sputum study, and TB treatment regimens could be frequently changed due to adverse effect of drugs and drug sensitivity test results. Fourth, as mentioned above, the accuracy and reliability of TB notification in North Korea and the adequacy of methods for further estimation, are under serious scrutiny. Therefore, greater cooperation with the WHO and non-government organizations active in North Korea needs to be established to enable access to more accurate and reliable TB data for North Korean residents. Lastly, we could not be assured of the originality of the infected strains in NKDs, because of the absence of the standardized whole genome sequencing data (WGS) of the North Koreans, not to mention the WGS data of the NKD (35). This analysis would be beneficial to establish further public TB infection control strategies for all immigrants including the NKD who are supposed to be under severe stressful conditions and poor nutrition status during the evacuation process as well as to better understand strain diversity, transmission patterns, and the potential introduction of drug-resistant TB strains into South Korea.

## Data Availability

The data analyzed in this study is subject to the following licenses/restrictions: NHIS claims data is available under the permission of the National Health Insurance Service. Requests to access these datasets should be directed to https://nhiss.nhis.or.kr/.

## Supplementary material

SUPPLEMENTARY TABLE 1 Definition of KCD8 disease classification and drug code of pharmacy. KCD8, Korean standard classification of disease; TB, tuberculosis.

SUPPLEMENTARY FIGURE 1 Proportion of age groups in South Korean control group and North Korean defectors. NHIS, national health insurance service.

SUPPLEMENTARY FIGURE 2 Proportion of bacteriologically confirmed tuberculosis. NHIS, national health insurance service. TB, tuberculosis; WHO, World Health Organization.

SUPPLEMENTARY FIGURE 3 Treatment success rate of multidrug resistant tuberculosis in native South Korean and North Korean population. MDR-TB, multidrug-resistant tuberculosis; XDR-TB, extensive drug-resistant tuberculosis.
