# Supplementary material for: Discrepancies in tuberculosis burden estimates: North Korean defectors vs. official reports
Source: Front Public Health. 2025 May 30;13:1545628. doi: 10.3389/fpubh.2025.1545628 (PMC12163040; doi:10.3389/fpubh.2025.1545628)
Supplement: SUPPLEMENTARY TABLE 1 — Definition of KCD8 disease classification and drug code of pharmacy. KCD8, Korean standard classification of disease; TB, tuberculosis. [file Table_1.docx]

**Supplementary material 1**. Definition of KCD8 disease classification and drug code of pharmacy.

| **TB Disease Codes** | **A15, A16, A17, A18, A19, P370, U843, U880, U881** |
| --- | --- |
|  |  |
| **Drug Names** | **Drug Codes** |
| Amikacin | 106803BIJ,106831BIJ |
| Bedaquiline fumarate | 626901ATB,223902ATB,223903ATB,223904ATB |
| Capreomycin | 448101BIJ |
| Ciprofloxacin(hydrochloride) | 134103ATB,134105ATR |
| Clarithromycin | 134901ATB,134902BIJ |
| Cycloserine | 139101ACH |
| Delamanid | 631101ATB,106833BIJ,106834BIJ,106836BIJ,106830BIJ,106835BIJ,106832BIJ,106801BIJ,,106804BIJ,106805BIJ |
| Ethambutol | 155602ATB,155605ATB |
| Isoniazid | 178101ATB |
| Isoniazid+rifampicin | 380200ATB,183235BIJ,183236BIJ,183201ATB,183202ATB,183205ATB,183235BIJ,183236BIJ,183203ATB,183202BIJ,183203BIJ,183205BIJ,183206BIJ |
| Isoniazid+rifampicin+ethambutol | 801601BIJ,380302BIJ,380303BIJ |
| Isoniazid+rifampicin+ethambutol+pyrazinamide | 519500ATB,203940BIJ,203902BIJ |
| Isoniazid+rifampicin+pyrazinamide | 489200ATB |
| Kanamycin | 179401BIJ |
| Levofloxacin(hydrate) | 183233BIJ,183234BIJ,208101AGN |
| Linezolid | 412901ATB,412903ATB,412930BIJ,412901BIJ |
| Meropenem | 190702BIJ,190704BIJ,134904ATB,134933ASY,134934ASY,134935ASY,134942ASY,134936ASY,134902BIJ,134904ATR,134937ASY,134939ASY,134901ATB,134937ASY,134904ATB,134940ASY,134930ASY,134931ASY,134903ASY,134905ASY |
| Moxifloxacin(hydrochloride) | 380335BIJ,380301ATB |
| Ofloxacin | 203901ATB,203904ATB |
| P-aminosalicylic acid (calcium p-aminosalicylate) | 208131AGN,208130AGN,190703BIJ |
| Prothionamide | 220401ATB |
| Pyrazinamide | 221201ATB,221202ATB |
| Rifabutin | 364401ACH |
| Rifampicin | 223901ACH,223902ACH,134108ATR,134109ATB, 134133BIJ,,34134BIJ,134103ATB,134135BIJ,134105ATB,134101BIJ,134104BIJ,134106BIJ,134103ACH |
| Streptomycin | 232101BIJ |
