# Supplementary figures and images for: Discrepancies in tuberculosis burden estimates: North Korean defectors vs. official reports
Source: Front Public Health. 2025 May 30;13:1545628. doi: 10.3389/fpubh.2025.1545628 (PMC12163040; doi:10.3389/fpubh.2025.1545628)

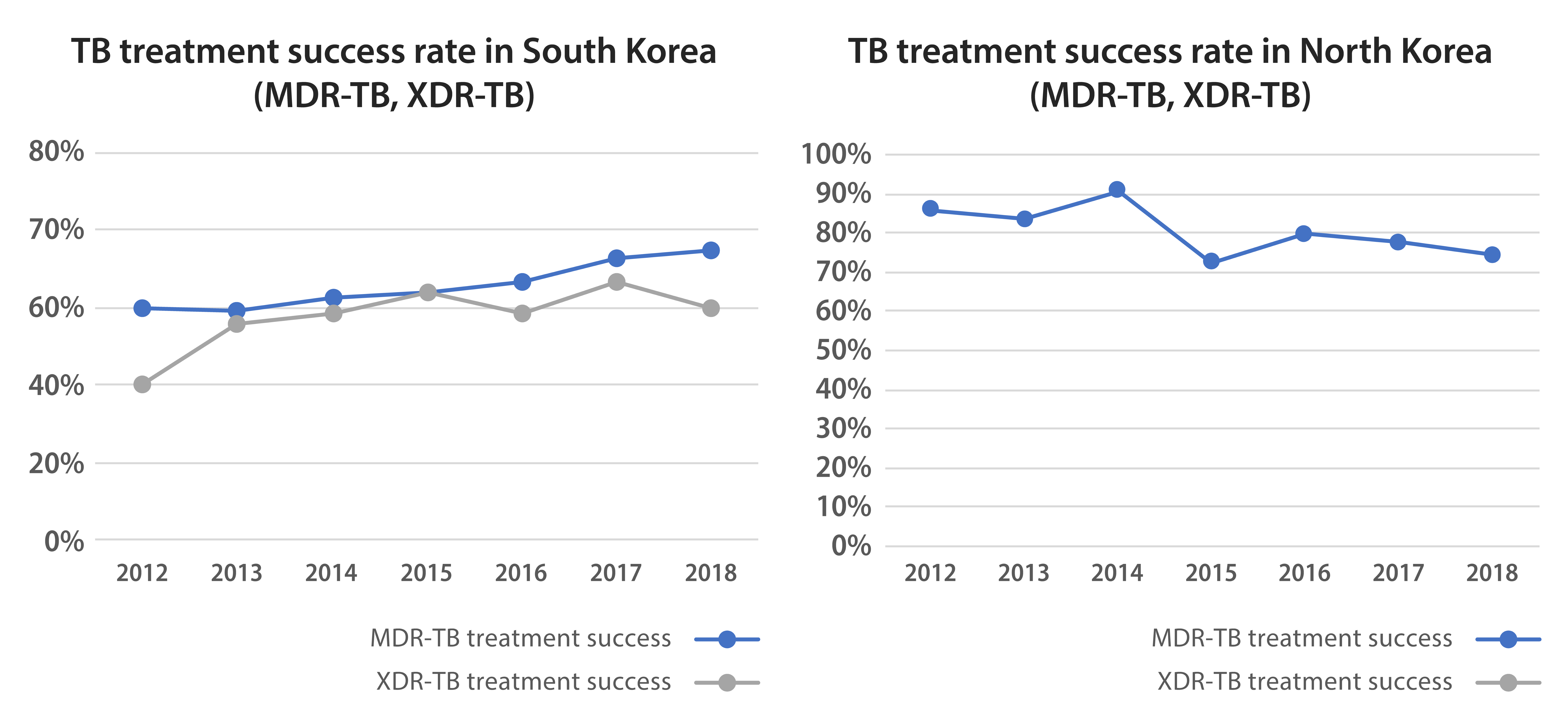

Supplement: SUPPLEMENTARY FIGURE 3 — Treatment success rate of multidrug resistant tuberculosis in native South Korean and North Korean population. MDR-TB, multidrug-resistant tuberculosis; XDR-TB, extensive drug-resistant tuberculosis. [file Image_3.tif]
